# The Effect of Fire Smoke Exposure on Firefighters’ Lung Function: A Meta-Analysis

**DOI:** 10.3390/ijerph192416799

**Published:** 2022-12-14

**Authors:** Joana V. Barbosa, Mariana Farraia, Pedro T. B. S. Branco, Maria Conceição M. Alvim-Ferraz, Fernando G. Martins, Isabella Annesi-Maesano, Sofia I. V. Sousa

**Affiliations:** 1 Laboratory for Process Engineering, Environment, Biotechnology and Energy (LEPABE), Faculdade de Engenharia, Universidade do Porto, Rua Dr. Roberto Frias, 4200-465 Porto, Portugal; 2 ALiCE—Associate Laboratory in Chemical Engineering, Faculty of Engineering, University of Porto, Rua Dr. Roberto Frias, 4200-465 Porto, Portugal; 3 Desbrest Institute of Epidemiology and Public Health (IDESP), Institut National de la Santé et de la Recherche Médicale (INSERM), Montpellier University, 34093 Montpellier, France

**Keywords:** firefighters, lung function, meta-analysis, occupational exposure, FVC and FEV_1_

## Abstract

Firefighters are exposed to a range of harmful substances during firefighting. Exposure to fire smoke has been associated with a decrease in their lung function. However, the cause–effect relationship between those two factors is not yet demonstrated. This meta-analysis aimed to evaluate the potential associations between firefighters’ occupational exposure and their lung function deterioration. Studies were identified from PubMed, Web of Science, Scopus and Science Direct databases (August 1990–March 2021). The studies were included when reporting the lung function values of Forced Expiratory Volume in 1 s (FEV_1_) or Forced Vital Capacity (FVC). The meta-analyses were performed using the generic inverse variance in R software with a random-effects model. Subgroup analysis was used to determine if the lung function was influenced by a potential study effect or by the participants’ characteristics. A total of 5562 participants from 24 studies were included. No significant difference was found between firefighters’ predicted FEV_1_ from wildland, 97.64% (95% CI: 91.45–103.82%; I^2^ = 99%), and urban fires, 99.71% (95% CI: 96.75–102.67%; I^2^ = 98%). Similar results were found for the predicted FVC. Nevertheless, the mean values of firefighters’ predicted lung function varied significantly among studies, suggesting many confounders, such as trials’ design, statistical methods, methodologies applied, firefighters’ daily exposure and career length, hindering an appropriate comparison between the studies.

## 1. Introduction

Exposure to fire smoke represents a severe health risk and is a growing concern for occupational and community exposures [1]. Fire smoke produces different compounds that are released into the environment, namely, particulate matter, water vapour and organic and inorganic gases such as carbon monoxide, nitrogen dioxide, polycyclic aromatic hydrocarbons, formaldehyde, benzene and acrolein [2,3]. These emissions depend on the region and fuel or the type of burning material, such as wood, plastics, chemical compounds (paints, solvents, pesticides and other chemicals) and oils.

In general, individuals exposed to fire smoke can undergo different health effects, both physical and mental, with varying severity levels. Some of the hazards and concerns for public health are airway and eye hypersensitivity, changes in vascular, pulmonary and cardiopulmonary function, different types of cancer and, in more severe cases, death [4,5,6,7,8,9,10].

The most vulnerable groups of the population include children, the elderly, pregnant women, individuals diagnosed with chronic cardiopulmonary diseases and occupational groups, especially emergency workers such as firefighters (including volunteers), police, rescue workers and health workers [9,11]. Firefighters, whose professional activity involves exposure to fire smoke, are a particular risk group because they might be exposed several times for extended periods.

In an attempt to understand the impacts of fire smoke exposure on firefighters’ health, some studies evidenced that fire smoke may cause pulmonary embolism, pneumonia, bronchitis, chronic obstructive pulmonary disease (COPD) and asthma, as well as their exacerbations [12,13,14,15]. Some chronic pulmonary diseases, namely, COPD, emphysema and chronic bronchitis, tend to increase airway resistance to expiratory airflow, leading to variations in FEV_1_ and FVC [16]. However, in some cases, these chronic respiratory diseases have been associated with patient’s age, smoking habits, occupation or metabolic disorders, such as diabetes [17,18]. On the other hand, some studies indicated that firefighters have superior lung function than the general population and attribute it to the regular use of self-contained breathing apparatus and the strong “healthy worker effect” [19,20,21]. This effect is usually seen in observational studies of occupational exposures and reflects that an individual must be healthy to be employable in a workforce [22,23].

The majority of those studies in the literature described urban fires, mostly related to the collapse of the World Trade Centre (WTC) on 11 September 2001 (9/11) [24,25,26], while others were related to wildland fires, prescribed [27,28,29,30] or not [13,31,32,33]. Still, comparisons correlating those types of fires with the lung function of firefighters are lacking. In addition, given the above-referred inconsistency in the association of firefighters’ lung function reduction and firefighting exposure, a comprehensive perspective of this impact should be achieved.

As far as known, only systematic reviews have been published in this area. Thus, this study aimed to perform a meta-analysis to understand and quantify the impact of fire exposure on firefighters’ pulmonary health.

## 2. Materials and Methods

### 2.1. Search Strategy

Four databases, namely, PubMed, Web of Science, Scopus and Science Direct, were accessed in March 2021, using the same search terms: “firefighter”, “health effects”, “spirometry”, “asthma”, “occupational exposure”, “obstructive airway”, “lung”, “FEV”, “forest”, “wildland”, “chronic obstructive pulmonary disease” and “meta-analysis”. References cited in individual or review articles were systematically analysed through a manual search and included in this meta-analysis. No language limitation was applied. Duplicates were removed. The authors were not contacted for further information. This meta-analysis followed the PRISMA (Preferred Reporting Items for Systematic Reviews and Meta-Analyses) guidelines [34].

### 2.2. Study Design and Eligibility Criteria

Only cohort or case–control studies, which specifically evaluated the lung function and reported FEV_1_ and FVC values, were included. The primary search results were reviewed, and some of the articles were eliminated after reviewing their title and abstract.

The applied criteria for exclusion of a study were: (i) not related to lung function; (ii) not related to firefighter workers; (iii) not related to fire exposure; (iv) not reporting FEV_1_ or FVC; (v) overlapping study population; and (vi) published in books/book chapters, reviews, textbooks and reports.

### 2.3. Data Extraction

The studies were independently researched and screened by two authors. Agreement between them was reached after a consensual discussion. For studies considering the same or overlapping populations, we selected the one with a larger population and more comprehensive information.

After applying the exclusion criteria, the studies were analysed, and data were extracted with the following information: author’s surname, publication year, study design, study location, sample size, participants’ mean age, fire type and main objective. Predicted FEV_1_ (%) and FVC (%) were collected as indicators of lung function. When FEV_1_ and FVC were expressed in litres, the corresponding predicted values were estimated using the Global Lung Function calculator, which is based on the age, gender, height and ethnicity of the study participants [35].

The missing standard deviations (SD) data were calculated from trial statistics, namely, confidence intervals and standard errors of the mean [36].

### 2.4. Statistical Analysis

Meta-analyses of the lung function values were carried out using the generic inverse variance in R software version 3.6.1 (R Foundation for Statistical Computing, 2019), with the meta package [37]. Data analyses were pooled using a random-effects model since there was an important statistical heterogeneity between the trials and also because some groups had few data points, and it was necessary to estimate the group’s effect based partially on the most abundant data of other groups [36]. Heterogeneity between studies was evaluated using I square statistic (I^2^), Tau squared (τ^2^) and the standard chi-squared test (χ^2^). I^2^ is the proportion of the dispersion of the results observed in the studies included in a meta-analysis that is real, rather than specious. The I^2^ index can be considered as the percentage of the total variability in a set of effect sizes due to true heterogeneity (variability between studies). τ^2^ is the variance of the effect size parameters between the populations of studies, reflecting the variance of the true effect sizes. χ^2^ tests the statistical hypothesis that the true treatment effects (the parameters of effect size) are the same in all the primary studies included in a meta-analysis [38]. Heterogeneity was considered high for I^2^ values > 75%, moderate for values between 50% and 75% and low for values < 25%. The level of statistical significance was set at 0.05.

Subgroup analyses were used to determine if the lung function was influenced by a potential effect of the study or by participants’ characteristics, such as publication year (before 1996, between 1997 and 2006, 2007 and 2013 and after 2014), study location (Europe, North America, Asia and Australia), participants’ age (20–30, 31–40 and more than 41 years of age), smoking practices (non-smokers and smokers) or type of fire (wildland or others).

For the subgroup analysis by smoking practices, the studies were divided according to the number of smoking participants included in each study, with “non-smokers” and “smokers” assigned to the studies wherein less than or equal to 10% of the participants were non-smokers and more than 10% of the participants were smokers, respectively. Hereafter, whenever these terms are mentioned, it is intended to be understood as smoking and non-smoking participants.

### 2.5. Risk of Bias Assessment

The methodological quality and the risk of bias for each of the included studies were assessed by two independent authors based on the Study Quality Assessment Tools [39]. This tool was adapted to use in this meta-analysis and consists of 14 questions covering the following domains: study objective, study population, sample definition and selection, interventions/exposure, outcomes, reference equations, confounding variables and statistical methods. The possible responses were “Yes”, “No” or “N.A.” (not applicable). The risk of each study was scored as “Low” (L, [0.00–0.40]), “Moderate” (M, [0.40–0.70]) or “High” (H, [0.70–1.00]). Disagreements between the two authors were overcome after discussion.

## 3. Results

### 3.1. Literature Search and Study Characterisation

**Figure 1:**
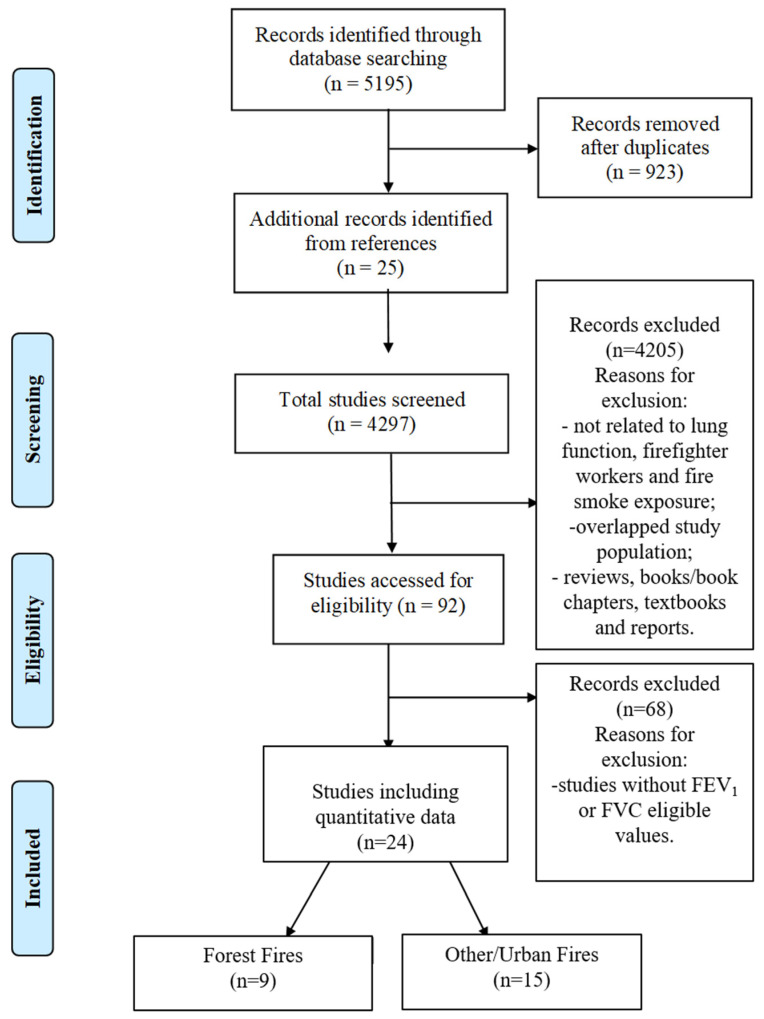
PRISMA flow diagram describing the search and selection procedures of the meta-analysis.

After removing duplicates and including the records identified through manual reference analysis, the literature search identified 4297 studies. After screening the titles and abstracts, 4205 studies were excluded according to the exclusion criteria. Regarding specifically the criterion of overlapping study populations, several studies related to the WTC fire were found. However, as the analyses were performed based on the same population of firefighters exposed to the pollution of the WTC fire, only one study (the most complete) was considered in this meta-analysis. After reading the full text of the 92 remaining studies, 68 more were excluded, since the FEV_1_ values were not available or were not eligible. Finally, the 24 studies obtained were divided into wildland (9 studies) and others/urban fires (15 studies). The PRISMA flow diagram is provided in Figure 1.

Table 1 lists the selected studies and summarises their main characteristics. All of them were published in the last 31 years, but the majority were published between 2007 and 2018 (15 studies, 71%). Most of the studies included in this meta-analysis had cross-sectional (63%) designs, although there were some with cross-shift (25%) and cross-season (12%) designs. The studies were performed in Europe (11), the United States of America (10), Australia (2) and Asia (1). The participants’ age varied between 20 and 30 years (30%), 31 and 40 years (35%) and above 41 years (35%), and the participants were all professionals, excluding those from Portugal [40], that included volunteers. Considering the smoking practices, half of the studies included smoking participants (48%), and the remaining (52%) evaluated non-smoking participants.

### 3.2. Lung Function Data

Table 2 summarises the results obtained in the pooled analysis. The predicted FEV_1_ mean values reported in the studies included in this meta-analysis varied from 82.94% to 113.39%. The overall predicted FEV_1_ mean value obtained for the 24 studies was 99.23% (95% CI: 94.65–103.80%), with a heterogeneity of I^2^ = 100% (Supplementary Figure S1). On the other hand, the predicted FVC mean values reported in all the included studies ranged from 83.68% to 121.76%. The overall predicted FVC mean value was 103.08% (95% CI: 99.83–106.32%), with a heterogeneity of I^2^ = 99% (Supplementary Figure S2).

#### 3.2.1. Sub-Group Analysis

**Figure 2:**
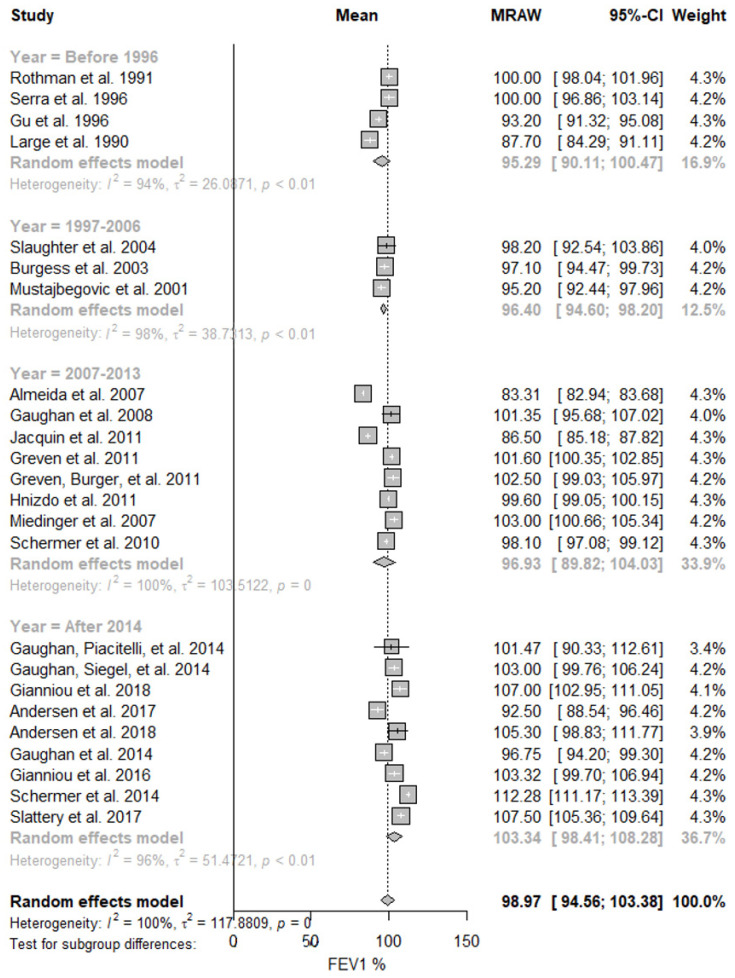
Predicted FEV_1_ in firefighters stratified by studies’ publication year. Forest plot displaying the weight of the predicted FEV_1_ mean value and heterogeneity [13,40,41,42,43,44,45,46,47,48,49,50,51,52,53,54,55,56,57,58,59,60,61,62].

Figure 2 shows the results obtained for firefighters’ lung function stratified by publication year. The meta-analysis showed that the predicted FEV_1_ mean value increased from 95.29% (95% CI: 90.11–100.47%; I^2^ = 94%) in the studies performed before 1996, to 103.34% (95% CI: 98.41–108.28%; I^2^ = 96%) in the studies published after 2014. This evident increase over the years was also observed in the predicted FVC mean values (Table 2).

**Figure 3:**
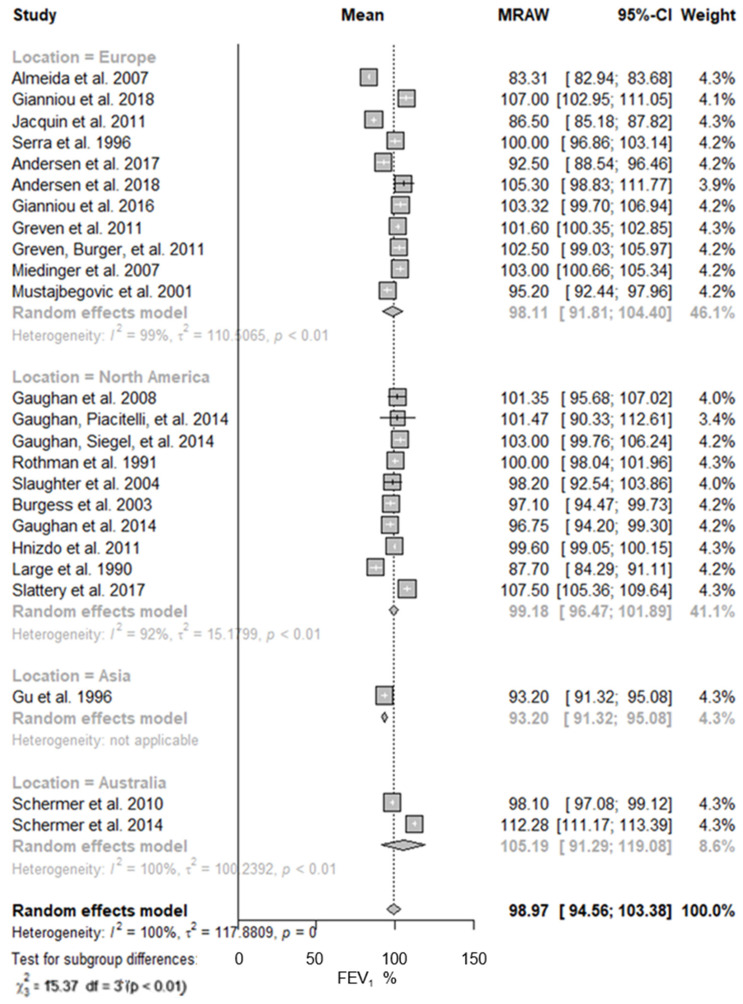
Predicted FEV_1_ in firefighters stratified by study location. Forest plot displaying the weight of the predicted FEV_1_ mean value and heterogeneity [13,40,41,42,43,44,45,46,47,48,49,50,51,52,53,54,55,56,57,58,59,60,61,62].

When stratified by study location, the predicted FEV_1_ values were slightly different (Figure 3). Although a single study performed in Asia was included in this meta-analysis, its participants showed the lowest predicted mean value of FEV_1_, 93.20% (95% CI: 91.32–95.08%; I^2^ = n.a.), while the participants from Australia showed the higher mean value of lung function, 105.19% (95% CI: 91.29–119.08%; I^2^ = 100%). There were no significant differences between the participants from Europe, 98.11% (95% CI: 91.81–104.40%; I^2^ = 99%), and those from North America 99.18% (95% CI: 96.47–95.08%; I^2^ = 92%). The results obtained for the predicted mean values of FVC with the participants from Australia were also the highest, 111.10% (95% CI: 92.28–129.91%; I^2^ = 100%); however, the FVC values of the study performed in Asia, 103.90% (95% CI: 101.78–106.02%; I^2^ = n.a.), were slightly higher than those registered both in Europe, 102.65% (95% CI: 98.92–106.39%; I^2^ = 94%), and in North America, 101.60% (95% CI: 97.46–105.74%; I^2^ = 99%) (Table 2).

**Figure 4:**
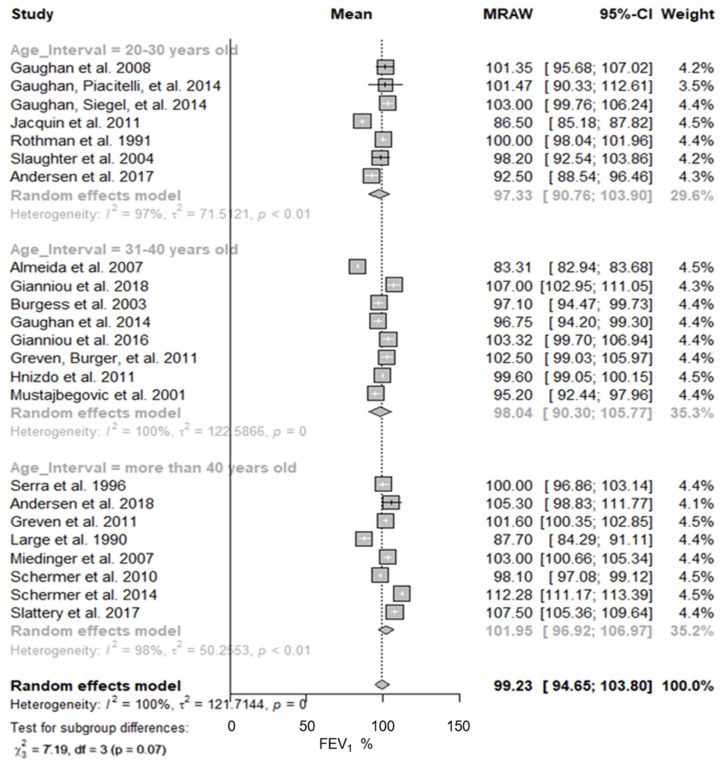
Predicted FEV_1_ in firefighters stratified by participants’ age. Forest plot displaying the weight of the predicted FEV_1_ mean value and heterogeneity [13,40,41,42,43,44,45,46,47,48,49,50,51,52,53,54,56,57,58,59,60,61,62].

Figure 4 shows the predicted FEV_1_ in firefighters according to their age. The youngest individuals (groups 20–30 and 31–40 years old) showed similar predicted FEV_1_ mean values (97.33% and 98.04%, respectively), while the oldest participants (older than 40 years) showed a higher predicted mean value of FEV_1_, 101.95% (95% CI: 96.92–106.97%; I^2^ = 98%). The same behaviour was observed for the predicted mean values of FVC (Table 2).

**Figure 5:**
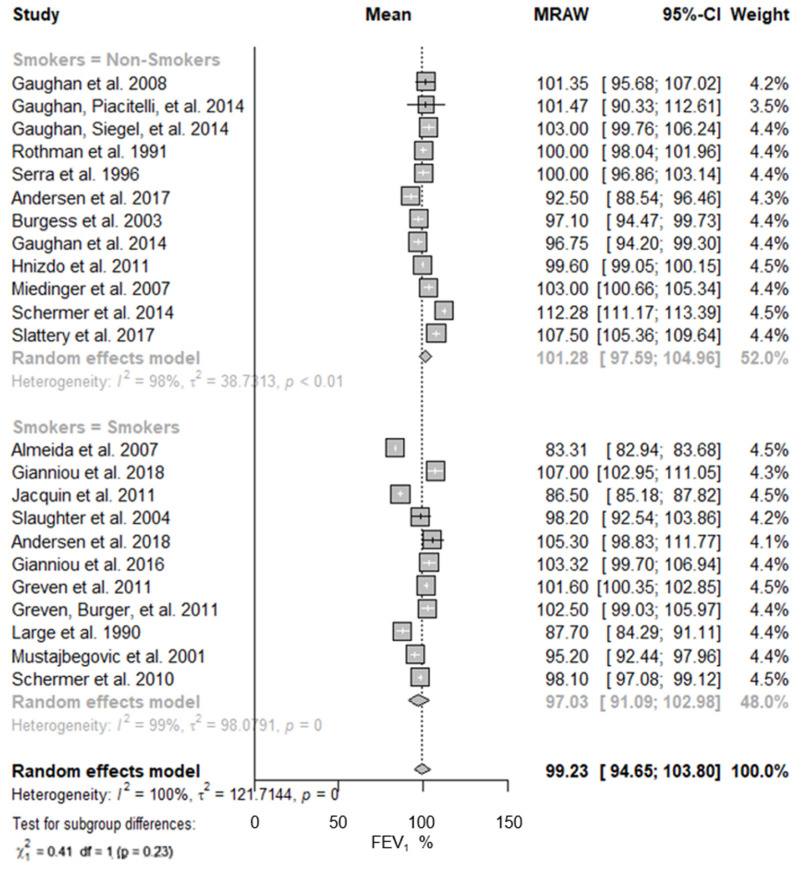
Predicted FEV_1_ in firefighters stratified by smoking practices. Forest plot displaying the weight of the predicted FEV_1_ mean value and heterogeneity. “Non-smokers” was assigned to all the studies wherein less than or equal to 10% of the participants were smokers, and “Smokers” was assigned to the studies wherein more than 10% of the participants were smokers [13,40,41,42,43,44,45,46,47,48,49,50,51,52,53,54,56,57,58,59,60,61,62].

Smoking practices showed that the lung function in non-smoker firefighters was higher than in smokers, being 101.28% (95% CI: 97.59–104.96%; I^2^ = 98%) and 97.03% (95% CI: 91.09–102.98%; I^2^ = 99%), respectively (Figure 5). Identical results were obtained for the predicted FVC mean values (Table 2).

**Figure 6:**
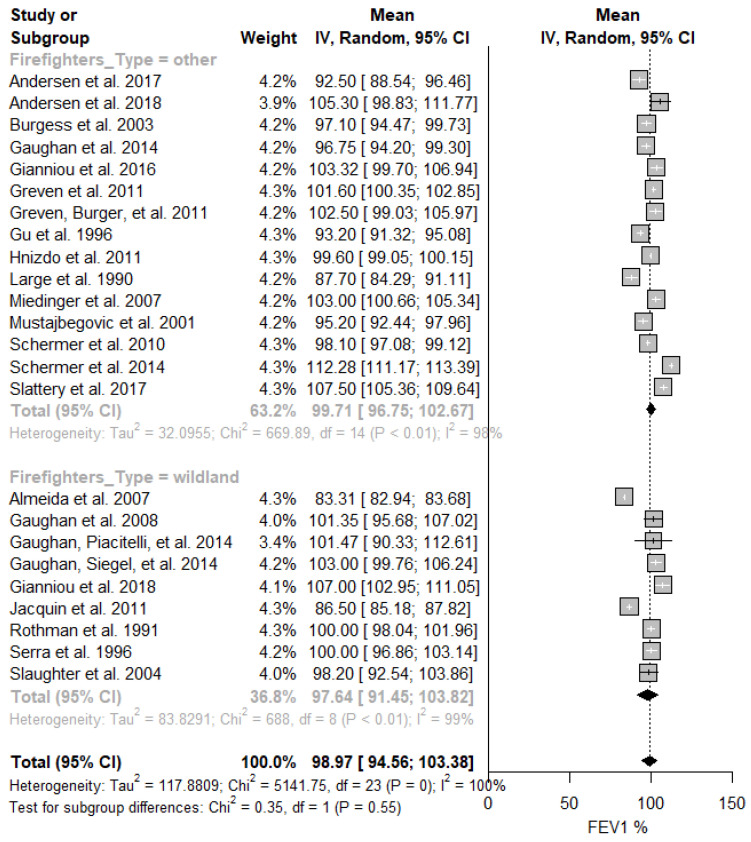
Predicted FEV_1_ in firefighters stratified by fire type. Forest plot displaying the weight of the predicted FEV_1_ mean value and heterogeneity [13,40,41,42,43,44,45,46,47,48,49,50,51,52,53,54,55,56,57,58,59,60,61,62].

In Figure 6, no significant differences were identified between firefighters that combat wildland fires (mean: 97.64%; 95% CI: 91.45–103.82%; I^2^ = 99%) and those fighting other/urban fires (mean: 99.71%; 95% CI: 96.75–102.67%; I^2^ = 98%). Similarly, there was no difference in the predicted FVC mean values (Table 2).

#### 3.2.2. Risk of Bias

Two articles were rated as low-quality, 14 studies as moderate-quality, and 8 studies as high-quality (Table S1). Considering the adapted scale used to evaluate the quality of individual studies, these generally failed to report the lung function as a predicted percentage and to report the *p*-values when assessing pre- and post-exposure. The most evident biases found were related to the study population, especially the justification for the sample size (only reported in two studies) and missing or unclear information about the participants’ selection (inclusion/exclusion criteria). Finally, only four studies defined control groups.

## 4. Discussion

The results from this meta-analysis were obtained from 24 studies conducted along 31 years, including studies with distinct designs, in various countries and considering different types of fires and firefighters of different ages and with different smoking practices. The results showed a large variability in the predicted FEV_1_ and FVC mean values. Moreover, no statistically significant difference was observed in the pooled predicted FEV_1_ mean value in firefighters (99.23%; 95% CI: 94.65–103.80%). This hindered definitive conclusions about the impact of firefighting exposure on the lung function. Still, the subgroup analysis allowed relevant interpretations.

Regarding the publication year, an increase in firefighters’ lung function along the years (in both predicted FEV_1_ and FVC values) was observed, although not statistically significant. However, the comparison between newer and older studies is difficult, as the studies’ procedures may have changed with time, namely, the recruitment strategies, the equipment used for lung function tests (past vs. modern devices), different or newer methodologies, or different standards for lung function tests [16,19]. Several studies based their conclusions on the published predicted values of spirometric indices (FEV_1_ and FVC), which have been reviewed or modified over the years. The Global Lung Function Initiative developed in 2012 (GLI-2012) reports the latest reference equations for spirometry, based on the pooled resources of various countries and on data obtained from more than 74,000 healthy non-smokers tests from all over the world [62,63]. To date, the GLI database is the one in force and is used by most of the researchers and health care professionals, hampering an appropriate comparison between studies.

Although non-statistically-significant differences were observed between different studies’ location, low pooled predicted FEV_1_ mean values were registered, except for Australian firefighters (105.19%; 95% CI: 91.29–119.08%). Schermer et al. (2010) and Slattery et al. (2017) also reported high FEV_1_ values and associated them with the “healthy worker effect”. This effect describes the reduction of morbidity or mortality associated with employment factors when occupational cohorts and the general population are compared [23,64]. In addition, the “healthy worker survivor effect” will contribute to confounders. This is influenced by the fact that, at the time of hiring, only physically fit workers are hired (healthy hiring), whereas people with health problems or with personal unhealthy habits and physical conditioning, such as high weight, alcohol consumption or smoking, are excluded [22]. On the other hand, healthy workers tend to stay in the workforce, but over time, the workers’ health status declines, and they leave the workforce. Several studies reported that in Australia the selection process to become a firefighter demands undergoing physical and psychological health and fitness tests and, once accepted, the selected individuals have to perform regular and intensive medical examinations. In addition, if they are not sufficiently healthy and fit, they are excluded and cannot proceed in their firefighter career [60,62].

The lung function did not significantly vary with the firefighters’ age. It is known that with age, individuals undergo anatomical and physiological changes (namely, after the age of 20–25 years), which are responsible for the reduction in their lung function [65]. Schermer et al. (2013) observed an increase in the lung function of younger generations of firefighters relative to the older generations. However, this was not always observed in studies involving firefighters’ lung function assessment. Kales et al. (1997) did not find significant differences between younger and older firefighters [66]. Probably, this heterogeneity observed between studies occurred due to the selection criteria that firefighters are subjected to in order to enter the career, namely, being healthy and very fit for service, regularly using self-contained breathing systems, meeting the strenuous physical demands that come with the job, or a combination of these factors [21].

Smoking is a known significant confounder. A slight reduction in smoking firefighters’ FEV_1_ predicted mean values compared to those of non-smokers was observed, though not statistically significant. Jacquin et al.’s study (2011), which was the wildland study with the highest score in the quality rating/lowest risk of bias assessment (0.92), reported that firefighters are likely to develop respiratory impairments after fire smoke exposure; however, the authors did not observe any statistical differences between smokers and non-smokers.

The pooled analysis was unable to demonstrate a significant difference in the predicted FEV_1_ mean values of both wildland and urban firefighters. Although this meta-analysis included 5562 participants, only 693 were firefighters dedicated to wildland fires, which probably may have contributed to overestimating the results obtained. Otherwise, some studies from North America assessed the lung function of firefighters who participated in WTC rescue operations. The collapse of the WTC generated a high-intensity pollution discharge, including extremely high Particulate Matter (PM) concentrations, exposing the population to extremely hazardous physical and chemical pollutants [67]. After this tragedy, populations exposed to the WTC pollution, including firefighters, showed an increase in sarcoidosis, leading to a reduction in their lung function, which may have contributed to mask the results obtained [68]. On the other hand, and as previously discussed, the “healthy worker effect” and/or the selection criteria to which firefighters are subject to in order to enter the career may have contributed to this result.

Heterogeneity was always above 90%. Perhaps, other factors that were not taken into account by most of the authors in the studies reviewed may have affected the results of the individual studies. Those include the duration of the intervention, i.e., the period between the end of the exposure and the lung function assessment (spirometry), the type of fire, and issues directly related to firefighters, namely, their age, other types of daily exposure besides that of firefighting, the annual number of working days and the career length.

Other factors that may have contributed to not having found significant differences in lung function reduction was the use of respiratory protective equipment and firefighter’s type of career. The protective apparatus attempts to minimize the respiratory hazards which firefighters are exposed to, filtering particulates from the surrounding air or providing breathable air when working in oxygen-deficient or toxic atmospheres [69]. On the other hand, the career of firefighters, which may be professional or voluntary, may have an influence on the indicators of the lung function, either because they may be less involved in firefighting or due to confounding factors regarding their professional activities. Anyway, in this meta-analysis, only one study included volunteers, with a total of 203 [40]. Regarding the aim of this meta-analysis, the use of the equipment and the career type may have influenced the results, impairing the analysis of the effect of fire combustion on firefighters’ lung function.

Therefore, the results of this meta-analysis should be interpreted with caution due to the potential heterogeneity between the trials.

### Limitations

There was significant heterogeneity in the studies’ aims, designs, statistical methods and methodologies applied, which can influence the quality of the results or lead to variations in the reported values of the lung function parameters (FEV_1_ and FVC).

The use of the GLI-2012 equations to transform the FEV_1_ and FVC values in litres into percent predicted values (%), and vice-versa, may have introduced bias in this study. Nevertheless, this is the most used methodology allowing an appropriate comparison between studies.

Further, due to the differences observed in how the data were reported, particularly, data measured in different units due to the use of different methodologies or data published in graphical figures, impairing the data extraction, it was necessary to exclude some studies or to perform recalculations (whenever possible) through the equations. Thus, this fact may have contributed to reduce the accuracy of the results.

## 5. Conclusions

The present study’s goal was to assess the potential associations between firefighters’ occupational exposure to fire smoke and its effect on the lung function. The large variability observed in the reported predicted FEV_1_ rates (between 82.94 and 113.39%) hindered definitive conclusions. Further, the pooled analysis was unable to demonstrate a statistically significant difference in firefighters’ predicted FEV_1_ mean values. Several factors could have contributed to this result, such as the methodologies applied, the equipment used, the reference equations for spirometry (Global Lung function Initiative–2012), the differences in the recruitment strategies of firefighters according to their countries or the publication year, hampering an appropriate comparison between studies.

Several reasons for statistical heterogeneity were identified. Since the results of the studies included in this meta-analysis may not have taken into account the possible confounding factors, they should be interpreted with caution due to the potential heterogeneity between the trials.

Although there are some limitations, this study’s conclusions are of upmost importance because they highlight the necessity for further studies to assess firefighters’ lung function, especially in those combating wildland fires. This will allow to understand the impacts of fire on firefighters’ health, which are still unknown, and develop strategies to protect them. Moreover, further development could also include an analysis of the composition of the pollutants in each study, enabling to understand if the fire smoke composition influences firefighters’ lung function reduction.

## Figures and Tables

**Table 1:** Main characteristics of the studies included in the meta-analysis.

Study	Design	Location	Sample Size	Mean Age (Years)	Main Objective
Wildland fires					
Almeida et al. (2007) [40]	cross sectional	Portugal	203	37.5	To assess the lung function in active firefighters.
Gaughan et al. (2008) [13]	cross-shift	USA	58	26.0	To assess the acute respiratory effects experienced by firefighters.
Gaughan et al. (2014) [41]	cross-shift	USA	17	26.0	To characterise exposures of firefighters and examine their effects on lung function changes.
Gaughan et al. (2014) [42]	cross sectional	USA	38	29.0	To assess the association between exposure, oxidative stress, cardiorespiratory function and symptoms in firefighters.
Gianniou et al. (2018) [43]	cross season	Greece	60	32.4	To assess post-exposure respiratory health and inflammation in firefighters with acute exposure to forest fire smoke.
Jacquin et al. (2011) [44]	cross season	Corsica	108	24.7	To evaluate the acute decline of the lung function and its persistence after a fire season in firefighters.
Rothman et al. (1991) [45]	cross season	USA	52	26.0	To evaluate the effects of firefighting on forced expiratory flow and respiratory symptoms.
Serra et al. (1996) [46]	cross sectional	Sardinia	92	40.9	To compare the respiratory function of firefighters with that of a control group.
Slaughter et al. (2004) [47]	cross-shift	USA	65	29.0	Short-term effects of exposures to fire smoke pollutants on the lung function of firefighters performing prescribed burns.
Other/Urban fires					
Andersen et al. (2017) [48]	cross-shift	Danish	53	21.4	To investigate the effect of firefighters’ activities on lung function, systemic inflammation and DNA.
Andersen et al. (2018) [49]	cross-shift	Danish	22	51.7	To investigate PAH exposure, lung function, systemic inflammation and DNA damage in firefighters after a day of work.
Burgess et al. (2003) [50]	cross sectional	USA	105	39.8	To evaluate biomarkers of lung injury resulting from occupational fire smoke exposure comparing firefighters and police officers.
Gaughan et al. (2014) [51]	cross sectional	USA	401	36.0	To assess the association between markers of systemic inflammation and lung function in firefighters.
Gianniou et al. (2016) [52]	cross sectional	Greece	92	30.0	To characterise airway and systemic inflammation in firefighters with a maximum occupational exposure of 1 year (trainees) compared to professional firefighters subjected to long-term exposure.
Greven et al. (2011) [53]	cross sectional	Netherlands	402	41.3	To determine associations between lung function, bronchial hyperresponsiveness and atopy with exposure to fire smoke among firefighters.
Greven et al. (2011) [54]	cross-shift	Netherlands	43	39.1	To determine associations between acute respiratory inflammatory responses, changes in bronchial hyperresponsiveness, serum pneumoprotein levels and exposure to fire.
Gu et al. (1996) [55]	cross sectional	Taipei	149	NA	To evaluate the health hazards of firefighters after fighting a fire which lasted for 40 h.
Hnizdo et al. (2011) [56]	cross sectional	USA	2043	39.2	To evaluate the impact of the intervention on the accuracy and precision of the lung function measurements and their estimated rate of decline.
Large et al. (1990) [57]	cross sectional	USA	60	42.0	To evaluate whether firefighters experience a significant change in spirometric values following exposure to smoke from a fire.
Miedinger et al. (2007) [58]	cross sectional	Switzerland	101	41.0	To assess professional firefighters’ respiratory health.
Mustajbegovic et al. (2001) [59]	cross sectional	Croatia	128	37.0	To determine the prevalence of chronic nonspecific respiratory diseases and of lung function abnormalities in firefighters.
Shermer et al. (2010) [60]	cross sectional	Australia	488	43.8	To establish if the use of impulse oscillometry reveals respiratory abnormalities in metropolitan firefighters that were not disclosed during routine screening with spirometry.
Schermer et al. (2014) [61]	cross sectional	Australia	570	46.63	To assess the prevalence of chronic respiratory conditions in metropolitan firefighters and to study associations between occupational exposure and use of respiratory protection devices with respect to health-related quality of life in firefighters with and without chronic respiratory conditions
Slattery et al. (2017) [62]	cross sectional	USA	212	46.4	To assess the validity of using the Global Lung Function Initiative’s (GLI) 2012 equations to interpret lung function data in a healthy workforce.

**Table 2:** Detailed results of the predicted FEV_1_ and FVC parameters obtained in the meta-regression.

	Pooled Random Effect Sizes					
	Predicted FEV_1_ (%)			Predicted FVC (%)		
	N (Studies)	Pooled Mean (95% CI)	Subgroup, *p*-Value	N (Studies)	Pooled Mean (95% CI)	Subgroup, *p*-Value
Total	24	99.23 (94.65; 103.80)	-	23	103.08 (99.83; 106.32)	-
Subgroup						
Publication year						
Before 1996	4	95.29 (90.11; 100.47)	0.07	4	98.10 (91.83; 104.37)	0.01
1997–2006	3	96.40 (94.60; 98.20)		3	99.85 (94.29; 101.40)	
2007–2013	8	96.82 (89.82; 104.03)		8	103.74 (101.23; 106.25)	
After 2014	9	103.34 (98.41; 108.28)		9	106.81 (100.03; 113.59)	
Location						
Europe	11	98.11 (91.81; 104.40)	0.01	11	102.65 (98.92; 106.39)	0.63
North America	10	99.18 (96.47; 95.08)		10	101.60 (97.46; 105.74)	
Asia	1	93.20 (91.32; 95.08)		1	103.90 (101.78; 106.02)	
Australia	2	105.19 (91.29; 119.08)		2	111.10 (92.28; 129.91)	
Age (years old)						
20–30	7	97.33 (90.76; 103.90)	0.49	7	100.24 (96.11; 104.38)	0.39
31–40	8	98.04 (90.30; 105.77)		8	103.51 (100.89; 106.13)	
More than 40	8	101.95 (96.92; 106.97)		8	104.12 (100.89; 106.13)	
Smokers						
Non-smokers	12	101.28 (97.59; 104.96)	0.23	12	104.07 (99.09; 109.05)	0.47
Smokers	11	97.03 (91.09; 102.98)		15	101.67 (97.73; 105.94)	
Fire type						
Wildland	9	97.64 (91.45; 103.82)	0.55	9	102.34 (98.29; 106.39)	0.74
Others/Urban	15	99.71 (96.75; 102.67)		15	103.30 (99.45; 107.15)	

## Data Availability

The datasets used and/or analysed during the current study are available from the corresponding author on reasonable request.

## Supplementary Materials

The following supporting information can be downloaded at: https://www.mdpi.com/article/10.3390/ijerph192416799/s1, Figure S1: Firefighters’ predicted FEV_1_ in the 24 studies analysed: forest plot displaying the heterogeneity and weight of the predicted FEV_1_ mean value [13,40,41,42,43,44,45,46,47,48,49,50,51,52,53,54,55,56,57,58,59,60,61,62]; Figure S2: Firefighters’ predicted FVC in the 24 studies analysed: forest plot displaying the heterogeneity and weight of the predicted FVC mean value [13,40,41,42,43,44,45,46,47,48,49,50,51,52,53,54,55,56,57,58,59,60,61,62]; Table S1: Summary of individual study quality/risk of bias assessment using Study Quality Assessment Tools. Studies are ordered by fire type and alphabetical order [13,40,41,42,43,44,45,46,47,48,49,50,51,52,53,54,55,56,57,58,59,60,61,62].
