# Supplementary material for: The Effect of Fire Smoke Exposure on Firefighters’ Lung Function: A Meta-Analysis
Source: Int J Environ Res Public Health. 2022 Dec 14;19(24):16799. doi: 10.3390/ijerph192416799 (PMC9779288; doi:10.3390/ijerph192416799)
Supplement: Supplementary file 1 [file ijerph-19-16799-s001.zip › ijerph-2051060-supplementary.pdf]

The Effect of Fire Smoke Exposure on Firefighters' Lung Function: A Meta-Analysis

Joana V. Barbosa [1,2], Mariana Farraia [1], Pedro T. B. S. Branco [1,2], Maria Conceição M. Alvim-Ferraz [1,2], Fernando G. Martins [1,2], Isabella Annesi-Maesano [3] and Sofia I. V. Sousa [1,2,*]

1 Laboratory for Process Engineering, Environment, Biotechnology and Energy (LEPABE)—Faculdade de Engenharia, Universidade do Porto, Rua Dr. Roberto Frias, 4200-465 Porto, Portugal
2 ALiCE—Associate Laboratory in Chemical Engineering, Faculty of Engineering, University of Porto, Rua Dr. Roberto Frias, 4200-465 Porto, Portugal
3 Desbrest Institute of Epidemiology and Public Health (IDESP), Institut National de la Santé et de la Recherche Médicale (INSERM), Montpellier University, 340093 Montpellier, France
* Correspondence: sofia.sousa@fe.up.pt

Supplementary Material

Study	Weight	Mean IV, Random, 95% CI
Almeida et al. 2007	4.3%	83.31 [82.94; 83.68]
Gaughan et al. 2008	4.0%	101.35 [95.68; 107.02]
Gaughan, Piacitelli, et al. 2014	3.4%	101.47 [90.33; 112.61]
Gaughan, Siegel, et al. 2014	4.2%	103.00 [99.76; 106.24]
Gianniou et al. 2018	4.1%	107.00 [102.95; 111.05]
Jacquin et al. 2011	4.3%	86.50 [85.18; 87.82]
Rothman et al. 1991	4.3%	100.00 [98.04; 101.96]
Serra et al. 1996	4.2%	100.00 [96.86; 103.14]
Slaughter et al. 2004	4.0%	98.20 [92.54; 103.86]
Andersen et al. 2017	4.2%	92.50 [88.54; 96.46]
Andersen et al. 2018	3.9%	105.30 [98.83; 111.77]
Burgess et al. 2003	4.2%	97.10 [94.47; 99.73]
Gaughan et al. 2014	4.2%	96.75 [94.20; 99.30]
Gianniou et al. 2016	4.2%	103.32 [99.70; 106.94]
Greven et al. 2011	4.3%	101.60 [100.35; 102.85]
Greven, Burger, et al. 2011	4.2%	102.50 [99.03; 105.97]
Gu et al. 1996	4.3%	93.20 [91.32; 95.08]
Hnizdo et al. 2011	4.3%	99.60 [99.05; 100.15]
Large et al. 1990	4.2%	87.70 [84.29; 91.11]
Miedinger et al. 2007	4.2%	103.00 [100.66; 105.34]
Mustajbegovic et al. 2001	4.2%	95.20 [92.44; 97.96]
Schermer et al. 2010	4.3%	98.10 [97.08; 99.12]
Schermer et al. 2014	4.3%	112.28 [111.17; 113.39]
Slattery et al. 2017	4.3%	107.50 [105.36; 109.64]
**Total (95% CI)**	**100.0%**	**98.97 [94.56; 103.38]**

Heterogeneity: $Tau^2 = 117.8809$; $Chi^2 = 5141.75$, $df = 23$ ($P = 0$); $I^2 = 100\%$

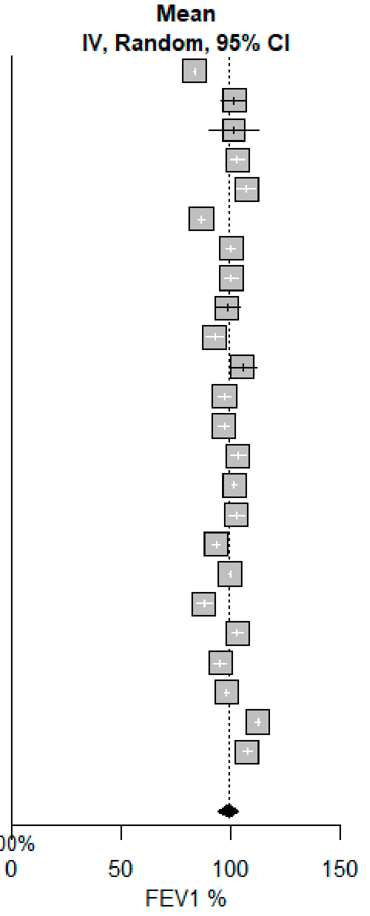

Figure S1. Firefighters predicted FEV_1 in the 24 studies analysed: forest plot displaying the heterogeneity and weighted of predicted FEV_1 mean value [13,40–62].

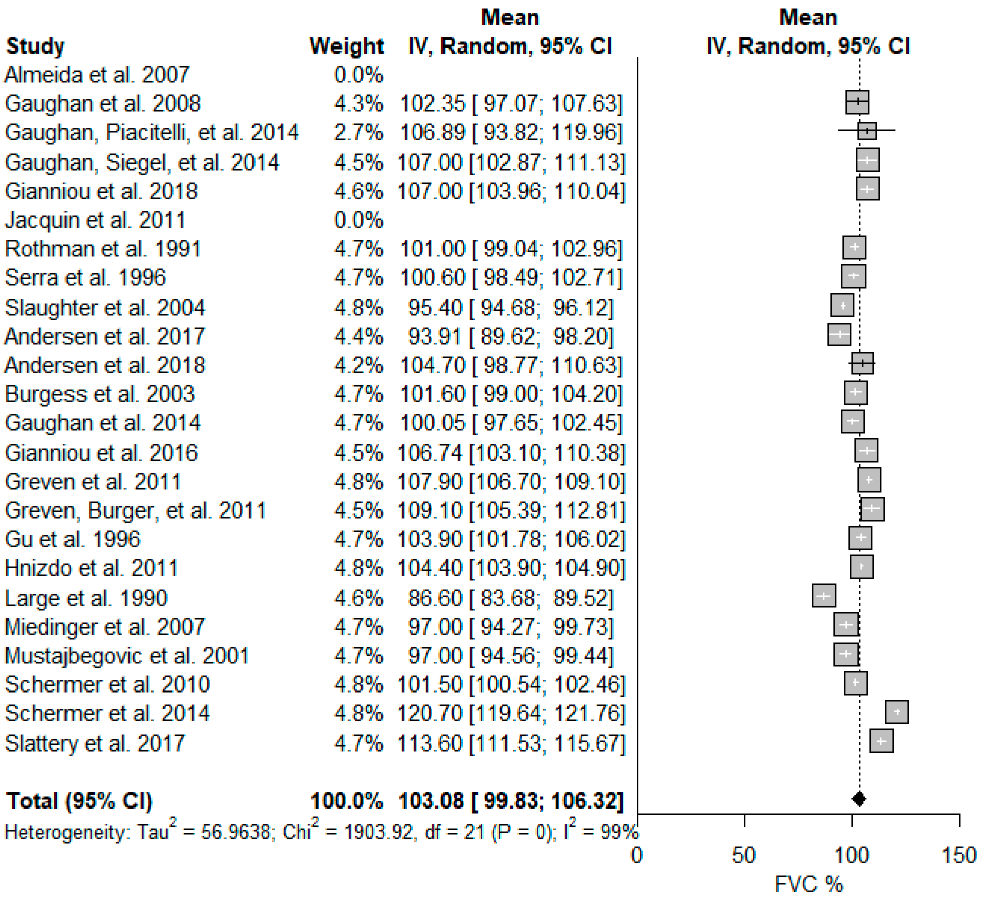

Figure S2. Firefighters predicted FVC in the 24 studies analysed: forest plot displaying the heterogeneity and weighted of predicted FVC mean value [13,40–62].

Table S1. Summary of individual study quality/risk of bias assessment using Study Quality Assessment Tools. Studies are ordered by fire type and alphabetical order [13,40–62].

	1	2	3	4	5	6	7	8	9	10	11	12	13	14	Score	Quality	Risk of Bias
Almeida et al., 2007	Yes	Yes	Yes	No	N.A.	No	Yes	No	No	No	N.A.	No	No	N.A.	0.36	L	H
Gaughan et al., 2008	Yes	Yes	No	No	Yes	Yes	Yes	Yes	No	No	Yes	Yes	Yes	N.A.	0.69	M	M
Gaughan, P., et al., 2014	Yes	Yes	No	Yes	Yes	Yes	Yes	Yes	Yes	No	Yes	Yes	No	N.A.	0.77	H	L
Gaughan, Siegel, et al., 2014	Yes	Yes	No	No	N.A.	No	Yes	Yes	No	No	N.A.	Yes	Yes	N.A.	0.55	M	M
Gianniou et al., 2018	Yes	Yes	Yes	No	No	Yes	Yes	No	No	No	Yes	Yes	Yes	N.A.	0.62	M	M
Jacquin et al., 2011	Yes	Yes	Yes	No	Yes	Yes	Yes	Yes	Yes	Yes	Yes	Yes	Yes	N.A.	0.92	H	L
Rothman et al., 1991	Yes	Yes	Yes	No	Yes	Yes	Yes	No	Yes	No	No	Yes	No	N.A.	0.62	M	M
Serra et al., 1996	No	Yes	No	No	N.A.	No	No	Yes	Yes	Yes	N.A.	No	Yes	Yes	0.50	M	M
Slaughter et al., 2004	Yes	Yes	No	No	Yes	Yes	Yes	No	Yes	No	Yes	Yes	No	N.A.	0.62	M	M
Andersen et al., 2017	Yes	Yes	Yes	No	Yes	Yes	Yes	No	Yes	No	Yes	Yes	No	N.A.	0.69	M	M
Andersen et al., 2018	Yes	Yes	No	No	Yes	Yes	No	No	Yes	No	No	Yes	No	N.A.	0.46	M	M
Burgess et al., 2003	Yes	No	No	No	N.A.	No	Yes	No	Yes	No	N.A.	Yes	Yes	Yes	0.50	M	M
Gaughan, C. et al., 2014	Yes	Yes	No	No	N.A.	No	Yes	Yes	Yes	Yes	N.A.	Yes	Yes	N.A.	0.73	H	L
Gianniou et al., 2016	Yes	No	No	No	N.A.	No	No	No	No	No	Yes	Yes	Yes	Yes	0.38	L	H
Greven et al., 2011	Yes	Yes	No	No	N.A.	No	Yes	Yes	Yes	Yes	N.A.	Yes	Yes	N.A.	0.73	H	L
Greven, Burger et al., 2011	Yes	Yes	No	Yes	Yes	Yes	Yes	Yes	No	No	No	No	Yes	N.A.	0.62	M	M
Gu et al., 1996	Yes	Yes	Yes	No	N.A.	No	Yes	No	Yes	Yes	N.A.	Yes	Yes	Yes	0.75	H	L
Hnizdo et al., 2011	Yes	Yes	N.A.	No	Yes	Yes	Yes	Yes	No	Yes	Yes	Yes	Yes	N.A.	0.83	H	L
Large et al., 1990	Yes	No	No	No	Yes	Yes	Yes	Yes	No	No	Yes	Yes	No	N.A.	0.54	M	M
Miedinger, et al., 2007	Yes	No	Yes	No	N.A.	No	Yes	Yes	No	Yes	N.A.	Yes	Yes	N.A.	0.64	M	M
Mustajbegovic et al., 2001	Yes	No	Yes	No	N.A.	No	Yes	Yes	Yes	Yes	N.A.	Yes	No	No	0.58	M	M
Schermer et al., 2010	Yes	Yes	Yes	No	N.A.	No	Yes	Yes	Yes	Yes	N.A.	Yes	Yes	N.A.	0.73	H	L
Schermer et al., 2014	Yes	Yes	Yes	No	N.A.	Yes	Yes	Yes	Yes	Yes	N.A.	Yes	Yes	N.A.	0.91	H	L
Slattery et al., 2017	Yes	Yes	Yes	No	N.A.	No	Yes	Yes	No	Yes	N.A.	Yes	No	N.A.	0.64	M	M

1 - Was the research question or objective in this paper clearly stated?; **2** - Was the study population clearly specified and defined (location, date, who…)?; **3** - Were all the subjects selected or recruited from the same or similar populations (including the same time period)? Were inclusion and exclusion criteria for being in the study prespecified and applied uniformly to all participants?; **4** - Did the authors include a sample size justification?; **5** - For the analyses in this paper, were the outcome(s) of interest measured prior and after to the exposure?; **6** - Was the outcome(s) assessed more than once over time?; **7** - Were the outcome measures (dependent variables) clearly defined, valid, reliable, and implemented consistently across all study participants?; **8** - Did the authors described which reference equations were used to calculate spirometric indices in % predicted? Did they report the criteria to select the best spirometric indices?; **9** - Were key potential confounding variables measured and adjusted statistically for their impact on the relationship between exposure(s) and outcome(s)?; **10** - Was the sample size sufficiently large to provide confidence in the findings?; **11** - Did the statistical methods examine changes in outcome measures from before to after the intervention? Were statistical tests done that provided p-values for the pre-to-post changes?; **12** - Were the statistical methods well-described?; **13** - Were the results well-described?; **14** - The study included controls? Did the authors reported, in the results section, the outcome result for the control group?

Legend	(grey)	- Yes	(black)	- No	(crossed box)	- N.A. (not applicable)
Quality rate / Risk of bias score	Low (L)	0.00 - 0.40	Moderate (M)	0.41 – 0.70	High (H)	0.71 – 1.0
